# Impact of a Mechanism-Based Anti-Aggression Psychotherapy on Behavioral Mechanisms of Aggression in Patients With Borderline Personality Disorder

**DOI:** 10.3389/fpsyt.2021.689267

**Published:** 2021-08-05

**Authors:** Hannah Honecker, Katja Bertsch, Karen Spieß, Marlene Krauch, Nikolaus Kleindienst, Sabine C. Herpertz, Corinne Neukel

**Affiliations:** ^1^Department of General Psychiatry, Medical Faculty, Center for Psychosocial Medicine, Heidelberg University, Heidelberg, Germany; ^2^Department of Psychology, Ludwig-Maximilians-University Munich, Munich, Germany; ^3^Institute of Psychiatric and Psychosomatic Psychotherapy, Central Institute of Mental Health, Medical Faculty Mannheim, Heidelberg University, Heidelberg, Germany

**Keywords:** aggressive behavior, borderline personality disorder, group psychotherapy, threat hypersensitivity, cognitive control, emotion recognition

## Abstract

**Introduction:** Aggressive behavior is highly prevalent in patients with borderline personality disorder (BPD) and represents a major burden for patients and their environment. Previous studies have hypothesized threat hypersensitivity, among other mechanisms, as a biobehavioral mechanism underlying aggressive behavior in patients with BPD. The effects of a 6-week mechanism-based anti-aggression psychotherapy (MAAP) for the group setting were tested in comparison to the effects of a non-specific supportive psychotherapy (NSSP) on this hypothesized mechanism and their relation to the effects on aggressive behavior.

**Methods:** To assess mechanisms of reactive aggression, 38 patients with BPD (20 in MAAP and 18 in NSSP) and 24 healthy controls participated in an emotion classification task before and after therapy or at a similar interval of 7 weeks for controls, respectively. In addition, current reactive aggressive behavior was assessed by the externally directed overt aggression score of the Overt Aggression Scale Modified (OAS-M) at both time points. Mixed linear models were used to test for group differences and differential treatment effects.

**Results:** Consistent with previous findings, patients showed longer response latencies and misclassified faces as angry more often than healthy controls. Comparing pre- and post-treatment measurements, the MAAP group showed an increase in response latency in classifying angry faces, whereas the NSSP group showed a decrease in latency. Furthermore, the difference between pre- and post-treatment response latencies in classifying emotional faces correlated with the reductions in reactive aggression in the MAAP group, but not in the NSSP group or healthy controls.

**Conclusion:** The results suggest an impact of MAAP on threat sensitivity as well as cognitive control, which has also been previously hypothesized as a biobehavioral mechanism underlying reactive aggression in patients with BPD. In addition, our findings shed light on the importance of these two biobehavioral mechanisms underlying reactive aggression as mechanisms of change addressed by MAAP. Further studies are needed to determine whether the behavioral change is stable over time and to what extent this change is related to a stable reduction in reactive aggression in a larger group of patients with BPD.

## Introduction

Aggression is a core feature of BPD (1) with over 70% of patients exhibiting aggressive behavior toward others within a year (2). Typically, aggressive behavior in BPD is classified as a form of reactive aggression characterized by impulsive and emotional behavior and triggered by real or perceived social threat, frustration, or social provocation (3). Several biobehavioral mechanisms of reactive aggression in BPD have been previously identified. Mancke et al. (4) proposed a multidimensional model according to which biobehavioral mechanisms including affective dysregulation, behavioral disinhibition, threat hypersensitivity, and reduced empathic functioning underlie reactive aggression in BPD. In this regard, a recent review by Bertsch et al. (5) on the brain mechanisms underlying reactive aggression distinguishes between strongly activating, bottom-up conditions such as threat hypersensitivity and poor regulatory, top-down conditions such as poor cognitive control, particularly underlying deficits in inhibitory control and emotion regulation.

Typically, common treatment programs for patients with BPD, such as dialectical behavior therapy [DBT, (6)] and mentalization-based therapy [MBT, (7)], focus on reducing emotional dysregulation, self-harm, and chronic suicidality (8) but do not specifically target aggressive behavior toward others (9). To address this gap, our group has developed an aggression-specific psychotherapeutic group intervention program called mechanism-based anti-aggression psychotherapy (MAAP) that aims to reduce aggressive behavior in patients with BPD by targeting the identified biobehavioral mechanisms of aggression in patients with BPD (4, 5). The rationale for targeting the proposed pathogenetic mechanisms underlying the specific psychopathology, namely the emergence of reactive aggression in patients with BPD, is that they can serve as therapeutic mechanisms of change (10) and thus mediate the therapeutic reduction of reactive aggression in patients with BPD. When tested against a non-specific supportive psychotherapy (NSSP), patients who participated in MAAP showed a clinically relevant 65% decrease in aggressive behavior according to the primary outcome *Overt Aggression Scale Modified* (OAS-M), compared with a 33% decrease in the NSSP group from the pre- to post-treatment time point. However, no significant difference in OAS-M overt aggression between MAAP and NSSP was found at post-treatment time point; at the 6-month follow-up time point, MAAP proved significantly superior to NSSP in reducing reactive aggression in BPD (9).

The focus of the present study was to examine the role of threat hypersensitivity as a mechanism of change in therapeutic reduction of reactive aggression. Threat hypersensitivity is characterized by an increased tendency to misclassify facial expressions as angry (11–14) and a hypervigilance to social threat cues (15), suggesting exaggerated bottom-up processing of high-salience social threat cues in BPD. Consistent with this conjecture, prolonged response latencies in response to threatening facial expressions have been discussed as indicative of difficulties in disengaging attention from these high-salience threat cues (16). Nonetheless, unlike eye movements, response latencies display rather late and cognitively controlled processes (17) and may also be interpreted as an expression of a top-down mechanism to regulate one's emotions when presented with high-salience threat cues (18).

In an emotion classification task displaying blends of angry and happy faces, patients with BPD showed an increased tendency to misclassify facial expressions as angry compared to healthy controls (13). Likewise, in a previous study by our group using an emotion classification task presenting angry, happy, fearful, or neutral faces, patients with BPD misclassified emotional or neutral faces as angry more often than healthy controls (19). These results suggest a biased perception of facial expressions as angry in patients with BPD.

Previous studies on emotion recognition of borderline patients differ with regard to their findings and assessment on response latency. Descriptively, some studies found overall prolonged reaction times in borderline patients compared to healthy controls, regardless of the emotion presented (16, 19). However, further studies also found a faster response of patients with BPD to the presentation of angry faces compared to controls (20) or comparable response times for angry faces and a significantly slower response to happy faces (13, 21), which may be a result of an increased detection ability of facial threat in patients with BPD (20). Thus, reaction times might also be influenced by detection thresholds in emotion recognition (19). Veague and Hooley (21) found that when presenting emotional expressions in increasing intensity, BPD predicted the earlier detection of anger. On the other hand, patients with BPD were less accurate in recognizing anger in faces when presented at full emotional intensity, which might be explained by increased arousal induced by threatening faces that impairs emotion classification (16). However, in further studies, no difference was found in the recognition of emotional expressions of different intensities in patients with BPD compared to healthy controls (12, 22). Despite previous findings regarding response latencies and detection threshold being rather scarce and heterogenous, they could hint at an exaggerated bottom-up processing of social threat cues in BPD (15). This could lead to both a lower detection threshold and shorter response latencies for angry facial expressions as well as difficulties in correct and fast emotion classification. We suppose that a reduction of this potentially heightened sensitivity for social threat cues following a therapeutic intervention could be displayed in a prolongation of response latencies when classifying angry faces.

In an emotion recognition study combining eye-tracking with functional neuroimaging, patients with BPD showed more and faster initial fixation changes to the eyes of angry faces accompanied by increased amygdala activation compared to healthy controls, which could represent threat hypersensitivity in BPD (20). In addition, hypervigilance to social threat cues might be particularly pronounced in aggressive patients with BPD, as self-reported trait aggressiveness in patients with BPD negatively correlated with the latencies of initial fixation changes to the eyes of angry and fearful faces, as well as the total fixation duration on angry eyes (19). However, in a study by Kaiser et al. (23), patients with BPD showed longer fixations of the eyes of different blends of emotional faces compared to non-patients, and thus no specific effect was found for the classification of angry faces. Another recent study assessing emotion classification in patients with BPD using eye-tracking also found faster initial fixation changes to the eyes by patients with BPD compared to non-patients regardless of emotional valence (24).

The aim of the present investigation was to assess the impact of MAAP on threat hypersensitivity as one of the proposed mechanisms of change in aggressive behavior in patients with BPD using an emotion classification task and eye-tracking. For this purpose, the task was performed before and after treatment with either MAAP or NSSP or at the same time interval in healthy volunteers. We hypothesized that (1) MAAP, but not NSSP, would reduce threat sensitivity as indicated by (a) a reduction of the proportion of misclassifications of emotional facial expressions as angry, (b) an increase in response latencies in response to angry faces, and (c) fewer and later saccades toward the eyes of angry faces in patients with BPD. Furthermore, we hypothesized that (2) change in threat sensitivity from pre- to post-treatment would correlate with reduction in aggressive behavior, namely the primary endpoint measured by the overt aggression scale of the OAS-M, in MAAP but not in NSSP or healthy controls.

## Methods

### Participants

A total of 38 patients with BPD participated in the study (20 in MAAP and 18 in NSSP). Additionally, 24 participants who had never fulfilled criteria for a psychiatric diagnosis or undergone any psychotherapeutic or psychiatric treatment were included as controls to relate the performance of patients with BPD in the emotion classification task, as well as potential changes therein, to the performance of participants without a current or lifetime psychiatric diagnosis. These participants are further referred to as healthy controls (HC). All participants had to be between 18 and 55 years old in order to be included. Further inclusion criteria for patients were meeting at least four BPD criteria according to DSM-IV (hence also including subthreshold BPD) and an overt aggression score (aggression score without auto-aggression) and irritability of at least 6 over a time span of 2 weeks, according to the *Overt Aggression Scale Modified* (OAS-M, see section Psychometric Measures). The group of healthy controls was matched to the MAAP and the NSSP group with regard to age and gender. In order to be included as healthy control the OAS-M overt aggression score and irritability score had to be <2. Exclusion criteria for all participants comprised pregnancy, neurological disorders, current substance abuse (except cannabis) or addiction as well as impaired vision (diopters ≥ ±1). Additional exclusion criteria for patients were additional non-study psychotherapy, bipolar I disorder or schizophrenia, as well as change in psychotropic medication within the last 3 weeks before allocation to trial. There was a dropout of five patients [two from MAAP (10.0%) and three from NSSP group (16.7%)] who did not start the treatment after participating in the behavioral laboratory measurements or discontinued treatment and did thus not participate in the post-treatment measurement. There was no dropout in the healthy control group. The behavioral data from the post-treatment measurements from four participants could not be recorded due to technical issues [one each from MAAP (5.0%) and HC (4.2%) and two from NSSP (11.1%)]. Consequently, 62 subjects participated in the pre-measurements (20 MAAP, 18 NSSP, and 24 HC) and 53 subjects (17 MAAP, 13 NSSP, and 23 HC) completed both measurements (pre- and post-treatment). The clinical characteristics of participants at the time of inclusion are illustrated in Table 1.

**Table 1:** Demographic and psychometric information of patients with borderline personality disorder (BPD, randomized into MAAP or NSSP treatment) and healthy controls (HC).

	**BPD** **(***n*****=** 38)**				**Group comparison**		**HC** **(***n*****=** 24)**		**Group comparison**	
	**MAAP** **(***n*****=** 20)**		**NSSP** **(***n*****=** 18)**		**MAAP vs. NSSP**				**BPD vs. HC**	
	**m (SD) or** ***n*** **(%)**		**m (SD) or** ***n*** **(%)**		***X^2^*** or ***t***	***P***	**m (SD) or** ***n*** **(%)**		***X^2^*** or ***t***	***P***
Gender (female)	15 (75.0)		12 (66.7)		0.320	0.572	17 (70.8)		0.000	0.985
Age	28.85 (8.91)		30.50 (7.80)		−0.604	0.549	28.42 (5.47)		0.632	0.529
IQ	97.10 (17.53)		98.41 (15.07)		−0.242	0.810	111.70 (11.61)		−3.597	0.001
OAS-M										
Total	62.85 (41.05)		44.39 (20.90)		1.717	0.095	0.71 (0.75)		7.678	<0.001
Overt aggression	38.00 (29.80)		28.94 (17.99)		1.118	0.271	0.54 (0.59)		6.475	<0.001
Self-injury	16.20 (30.20)		7.17 (12.52)		1.180	0.246	0 (0)		2.458	0.017
Irritability	6.95 (0.89)		7.11 (0.83)		−0.576	0.569	0.46 (0.51)		34.002	<0.001
Suicidal tendency	1.70 (1.17)		1.17 (1.15)		1.411	0.167	0 (0)		5.998	<0.001
Number of BPD criteria	6.80 (1.28)		5.59 (1.23)		2.922	0.006	0 (0)		22.057	<0.001
ZAN (total)	13.56 (5.22)		12.13 (3.20)		0.920	0.356	0.13 (0.46)		14.110	<0.001
Current psychotropic medication	7 (35.0)		8 (44.4)		0.354	0.552	0 (0)		12.497	<0.001
Antidepressants	6 (30.0)		6 (33.3)		0.049	0.825	0 (0)		9.398	0.002
Neuroleptics	3 (15.0)		3 (16.7)		0.020	0.888	0 (0)		4.195	0.041
Other	0 (0)		2 (11.1)		2.346	0.126	0 (0)		1.305	0.253
Number of comorbidities										
Current	1.72 (1.36)		1.27 (1.33)		1.047	0.303	0 (0)		4.958	<0.001
Lifetime	2.22 (1.31)		2.65 (1.69)		−0.525	0.603	0 (0)		8.554	<0.001
Comorbidities	Current	Lifetime	Current	Lifetime			Current	lifetime		
Major depressive disorder	4 (20.0)	13 (65.0)	4 (22.2)	13 (72.2)			0 (0)	0 (0)		
Dysthymia	3 (15.0)	–	0 (0)	–			0 (0)	–		
Alcohol addiction/ abuse	0 (0)	3 (15.0)	0 (0)	4 (22.2)			0 (0)	0 (0)		
Anxiety disorders	8 (40.0)	8 (40.0)	4 (22.2)	4 (22.2)			0 (0)	0 (0)		
OCD	1 (5.0)	1 (5.0)	1 (5.6)	2 (11.1)			0 (0)	0 (0)		
PTSD	5 (25.0)	5 (25.0)	6 (33.3)	7 (38.9)			0 (0)	0 (0)		
Somatization disorders	0 (0)	–	0 (0)	–			0 (0)	–		
Eating disorders	1 (5.0)	3 (15.0)	1 (5.6)	5 (27.8)			0 (0)	0 (0)		
ASPD	4 (20.0)	5 (25.0)	0 (0)	2 (11.1)			0 (0)	0 (0)		
AVPD	5 (25.0)	4 (20.0)	7 (38.9)	5 (27.8)			0 (0)	0 (0)		

*Groups (BPD and HC) were matched with regard to age and gender. OAS-M, Overt Aggression Scale Modified; ZAN, Zanarini Rating Scale for BPD; OCD, Obsessive-compulsive disorder; PTSD, Post-traumatic Stress Disorder; ASPD, Antisocial personality disorder; AVPD, Avoidant personality disorder*.

Participants were recruited between January 2016 and January 2019 by the central project of the KFO-256, a Clinical Research Unit funded by the German Research Foundation, investigating mechanisms of disturbed emotion processing in BPD. Ethics approval was provided by the Ethics Committee of the Medical Faculty of the University of Heidelberg. Written informed consent was obtained from all participants.

### Psychometric Measures

At the time of inclusion, BPD and co-occurring avoidant personality and antisocial personality disorders were assessed through the *International Personality Disorder Examination for DSM-IV* [IPDE; (25)] and co-occurring axis I disorders were assessed using the *Structured Clinical Interview for DSM-IV* [SCID-I for axis I diagnoses; (26)]. Aggressive behavior of participants over the last 2 weeks was assessed using the *Overt Aggression Scale Modified* [OAS-M; (27)], a semi-structured interview assessing frequency and severity of overt aggressive behavior. The OAS-M consists of three subscales: overt aggression (items 1-4), irritability (items 5-6), and suicidality (items 7-7b). As our study focused on aggressive behavior that is shown against others, the sum of the first three items from the subscale overt aggression, namely verbal attacks, assaults against objects, and assaults against others, with all three items having a minimum value of zero and no upper limit were used as measure of overt aggressive behavior. The fourth item, namely self-harming behavior, was not included since the assessed behavior is not directed toward others. The *Zanarini Rating Scale for Borderline Personality Disorder* [ZAN; (28)] was used to assess the severity of borderline symptoms over the last week.

### Treatments

The two different group therapies were parallelized with regard to number of sessions and duration. The group therapy sessions were conducted over a period of 6 weeks with two sessions per week. In the week prior to the group therapy start, each patient participated in a single session with the group therapist. Each group session lasted one and a half hour. In total, we ran 12 therapy groups (6 x MAAP and 6 x NSSP) with three to six patients and two therapists per group. One further randomized group did not take place due to high dropout prior to group therapy start [see (9) for information on the randomized-controlled trial].

### Mechanism-based Anti-aggression Psychotherapy (MAAP)

MAAP is a highly structured manualized group psychotherapy program combining selected techniques from evidence-based treatment programs for BPD such as DBT and MBT and a specific attentional bias modification training. It particularly targets mechanisms that were proposed as important factors contributing to reactive aggression in BPD namely social threat hypersensitivity, maladaptive anger regulation, approach rather than avoidance of social threat cues, low capacity to adequately mentalize the intentions, cognitions and emotions of others and excessive emotional imitation and contagion. Thus, the therapeutic aims include psychoeducation on models of reactive aggression, and the development of inhibition and emotion regulation strategies by training skills derived from DBT (6). In addition MAAP included two app-based exercises practiced between sessions to reduce attentional bias toward threatening social cues: A visual search exercise instructed to find the only friendly looking face in a crowd of frowning or at least neutral faces (29), and another to target hidden smiling faces instead of hidden threatening faces, thereby strengthening the perception of social safety cues. Furthermore, MAAP included several techniques taken from MBT (7). The contents of each group session are presented in Table 2 and described in detail by Herpertz et al. (9).

**Table 2:** Overview of interventions and respective targets of group therapy sessions of MAAP.

**Session**	**Interventions**	**Targets**
1	Explaining model of reactive aggression and identifying triggers	Model of reactive aggression
2	Explaining model of emotion regulation	Model of emotion regulation
	Monitoring emotions	Anger regulation
3	Identifying cognitive schemata	Anger regulation
	Model of acceptance and commitment	Anger regulation
4	Skills to improve the regulation of irritability, anger, and rage	Anger regulation
	Body-related/physical exercises	Anger regulation
		Attentional training
5	Beware of traps	Discrimination presence vs. past
6	Mindfulness exercises	Attentional training
7	Inner monologs evoked by images of social scenes	Mentalizing training
8-11	Mentalizing scripts of real-life situations and associated inner monologs	Mentalizing training
12	Closing session	-
Between sessions	App-based attentional tasks	Attentional training

### Non-specific Supportive Psychotherapy

The comparator treatment to MAAP was a non-specific supportive psychotherapy comprising the same number of individual and group sessions as MAAP and focusing on non-specific factors that are known to be important components of psychotherapies in general, such as psychoeducation, reflective listening, empathy, and focus on patients' resources.

### Emotion Classification Task and Data Acquisition

The emotion classification task used in the present study has been previously employed in studies including patients with BPD (19, 24). Participants are presented with a total of 160 faces and asked to classify the emotion shown on each face. A fixation cross is presented before each trial (2,000 ms) and after each trial with a varying duration (1,000-3000 ms) to avoid anticipation effects. The task is based on a 2 x 2 x 4-design with the factors presentation time (condition), initial fixation and emotional expression: Faces are presented either 150 ms (brief condition) or 5,000 ms (long condition), allowing an assessment of reflexive initial saccades that appear after face presentation offset (brief condition) as well as detailed scanning of facial features (long condition). Furthermore, to control for the focus of initial fixation, faces are shifted either downwards or upwards so that either the eye or the mouth region appears in place of the fixation cross. Finally, the presented faces differ in the emotional expression that is shown (angry, fearful, happy, and neutral).

The presented stimuli were selected pictures of male and female actors, each unambiguously displaying angry, fearful, happy, and neutral expressions, from the following established picture sets: the Karolinska Directed Emotional Faces [KDEF; (30)], the NimStim Face Stimulus Set (http://www.macbrain.org/resources.htm), Pictures of facial affect (31), and the FACES database (32). The faces were slightly rotated in order to align both pupils on the same imagined horizontal line and cropped with an elliptic mask to remove hair and ears. The pictures were converted into grayscale images and the cumulative brightness was normalized across pictures.

Including instruction, training, and breaks between condition blocks (each block lasts about 10 min), the task takes approximately 1 h. The behavioral data collected in the task includes the proportion of correct responses (i.e., correct emotion classification) as well as the response latencies in trials with correct emotion classification. Furthermore, we recorded eye-tracking data, namely the proportion and the latency of initial saccades.

The eye-tracking data was recorded with a 60-Hz monocular eye-tracking system (ViewPoint, Arrington Research, Scottsdale, AZ, USA). The stimuli were presented on an Eizo FlexScan S2202 display (47.5 × 30.0 cm) with a resolution of 1,680 × 1,050 pixels and a refresh rate of 60 Hz using the software Presentation (Version 18.0, Neurobehavioral Systems, Inc., Berkeley, CA, www.neurobs.com) for stimuli presentation and response recording. The distance between the screen and the head location of participants, that was fixed using a chin rest and a forehead bar, was 57 cm.

### Data Reduction

The processing of data was conducted with R [Version 3.6.3; (33)]. In order to only assess saccades that represent a basal reaction on the presented face, we excluded trials that contained blinks as well as trials with saccades with an eye movement > 1° occurring between −300 and 150 ms relative to face onset. In the next step, all saccades with an eye movement > 1° occurring between 150 and 1,000 ms relative to face onset that were directed toward either the eye or mouth region were identified and included in the analysis. Each condition by initial fixation by emotion combination was presented 10 times to each participant per time point. We calculated the mean value for each of the assessed behavioral and eye-tracking data grouped by condition by initial fixation by emotion. Additionally, the proportion of misclassifications was used to calculate the error types made by each participant regarding the emotional expression as which faces were misclassified.

### Statistical Analysis

Mixed Models were used for data analyses employing the R package *Lme4*. Prior to the analyses, examination of variables revealed highly skewed distributions of the proportion of misclassifications as well as of the error types, which showed good fits to negative binomially distributions. Thus, for the analyses of each of the assessed variables in the eye-tracking task, we either used a generalized linear mixed model with negative binomial distribution for the proportion of misclassifications as well as the error types or, respectively, a linear mixed model for the proportion and the latency of initial saccades as well as the response latency. One advantage of mixed models over repeated-measurement analyses of variance (ANOVA) is that all available data is included in the analysis as no listwise deletion is applied. Therefore, linear mixed models can provide a better estimate of the unbiased treatment effects or more precisely, the change in the assessed variables from pre- to post-treatment measurements. In case of significant effects found in the mixed models, we subsequently used Tukey's HSD tests as *post hoc* tests corrected for multiple testing. To assess threat sensitivity before treatment, we first analyzed differences in behavioral data as well as eye-tracking data between patients with BPD and healthy controls at baseline with models that included all interaction effects. Thus, we included group (BPD vs. HC) as a fixed effect as well as the within-subject factors condition (brief, long), initial fixation (eye region, mouth region), and emotion (angry, happy, fearful, neutral) as fixed effects.

To test our first hypothesis that MAAP would reduce threat sensitivity, the same modeling procedure was used in the comparisons between pre- and post-measurements, adding time point as an additional fixed effect and including treatment (MAAP, NSSP, HC) instead of group as a fixed effect. Additionally, to control for pre-treatment differences in overt aggression between treatment groups, that were present despite randomization, we included pre-treatment overt aggression score as a covariate in the comparisons between pre- and post-measurements. Since we specifically hypothesized MAAP to impact behavioral and eye-tracking data regarding angry facial expressions, we subsequently used separate mixed models for each facial expression for further detailed analyses of time by treatment interactions.

In all models, we included subject-specific intercepts as random effects with an unstructured covariance structure (being the best fitting). Model diagnostics included visual checks for normality and homogeneity of residual variance for linear mixed models and normal distribution of random effects for generalized linear mixed models. When *p* > 0.10, interactions were removed from each model in hierarchical order (34). To compare the complete with the adapted models, we used the Maximum Likelihood Method and compared models on the basis of the Akaike-Information-Criterion (AIC) and the Bayesian-Information-Criterion (BIC). The final models were modeled on the basis of the Restricted Maximum Likelihood method as it is more robust when the sample size is small. Regarding the latency of initial saccades, only a subsample of *n* = 18 healthy controls, *n* = 18 patients with BPD in the MAAP group and *n* = 14 patients with BPD in the NSSP group in the long condition as well as only *n* = 3 in each of the three treatment groups in the brief condition showed initial saccades toward the other major facial feature (eye or mouth region) in each of the condition by initial fixation by emotion combination. Since emotion recognition does not require initial saccades for emotion classification, these trials may not be classified as invalid [see (19)]. However, as the sample size regarding the recording of the latency of initial saccades was critically reduced in the brief condition, we only performed analysis of saccadic latencies in the long condition.

Finally, in order to examine the relation between treatment change in aggressive behavior and treatment change in variables assessed in the emotion classification task (Hypothesis 2), we calculated the difference in the overt aggression scores from pre- to post-treatment measurements as well as the difference in those variables from the emotion classification task which showed change from pre- to post-measurements in MAAP but not in NSSP or HC in the previous steps of our analysis. Subsequently, we performed correlational analyses between the calculated treatment changes.

## Results

### Hypothesis 1

To test our first hypothesis, proposing a reduction of threat hypersensitivity related to treatment with MAAP, we analyzed the change in behavioral and eye-tracking data from pre- to post-treatment. Descriptive statistics of behavioral and eye-tracking data are presented in the Supplementary Table 1.

Regarding (a) the proportion of misclassifications of facial expressions as angry, analyses did not yield significant interaction effects of time by treatment with regard to error types (*X*^2^_2_ = 0.058, *p* = 0.971) or the proportion of misclassifications (*X*^2^_2_ = 2.641, *p* = 0.267) with all participants showing a high emotion recognition accuracy at pre- and post-treatment measurements (M ≥ 90.1%, SD ≤ 1.2%). The subsequent examination of time by treatment interactions in separate models for each emotion also showed no significant results. Prior to treatment, a significant main effect of error type emerged (*X*^2^_3_ = 23.678, *p* < 0.001) with all participants misclassifying faces more often as angry or fearful than happy (both *p* < 0.01). In addition, both the mean numbers and standard deviations relating to misclassifications of faces as angry were descriptively higher in patients with BPD (*M* = 3.1, *SD* = 4.2) than healthy controls (*M* = 1.5, *SD* = 1.8), while the interaction of group by emotion did not yield significance (*X*^2^_3_ = 0.830, *p* = 0.842). However, we found a significant higher order interaction effect of group by emotion by condition (*X*^2^_3_ = 8.084, *p* = 0.044). *Post-hoc* pairwise comparisons revealed that patients with BPD significantly misclassified facial expressions more often as angry than as neutral in the long condition (*p* < 0.05) while this was not found in the brief condition or in the group of healthy controls. Hence, misclassifications of facial expressions as angry were especially prominent in patients, replicating previous findings of an increased tendency to misclassify facial expressions as angry in patients with BPD when presentation time allowed for detailed scanning of faces. However, our hypothesis that MAAP would impact this tendency to misclassify facial expressions as angry was not confirmed.

**Figure 1:**
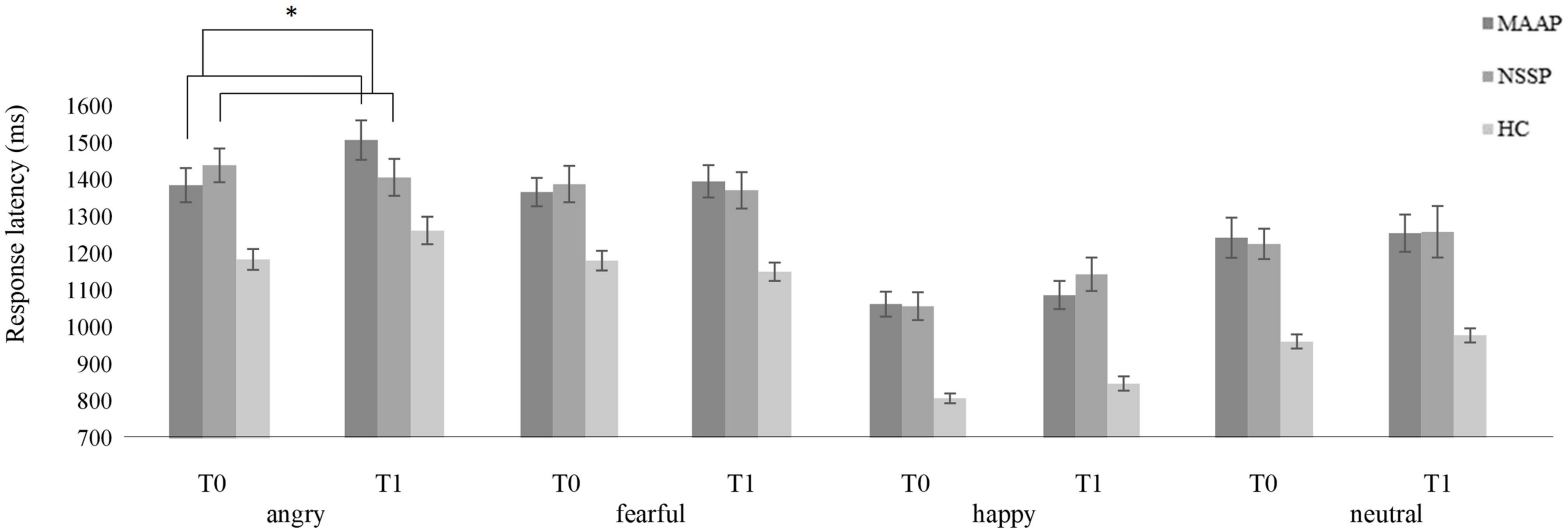
Mean response latency (± one standard error) of patients with BPD in the MAAP group, in the NSSP group and healthy controls when classifying emotional facial expressions (angry, fearful, happy, or neutral) at pre-treatment (T0) and post-treatment measurements (T1). Please note that only time by treatment interaction is highlighted; refer to text for further significant effects. *p < 0.05.

At the pre-treatment time point, we found a prolongation of (b) response latencies in response to all facial emotional expressions in patients with BPD [main effect of group (*F*_1, 60_ = 17.587, *p* < 0.001)] that did not change from pre- to post-treatment measurements [no significant time by treatment interaction (*F*_2, 52_ = 1.072, *p* = 0.350)], but the time by treatment by emotion interaction showed a non-significant statistical trend (*F*_6, 161_ = 1.948, *p* = 0.076). Analyzing the four emotions separately revealed a significant time by treatment interaction for the presentation of angry facial expressions (*F*_2, 52_ = 3.267, *p* = 0.046), while there were no interaction effects for the presentation of fearful (*F*_2, 52_= 1.553, *p* =.221), happy (*F*_2, 52_= 0.306, *p* = 0.738), or neutral facial expressions (*F*_2, 52_= 0.081, *p* = 0.922). In the subsequently conducted *post-hoc* test, we found that the MAAP group showed a prolongation of response latency when reacting to angry faces, that was significantly different to a shortening of response latency shown in the NSSP group from pre- to post-treatment measurements (*p* < 0.05). Thus, present findings are in line with our a priori hypothesis since we found that patients with BPD in the MAAP group showed a prolongation while the NSSP group showed a shortening in response latencies, a difference that only emerged when angry facial expressions were presented (see Figure 1).

With regard to (c) saccades measured by eye-tracking, the analyses did not reveal any significant effect of group (*F* ≤ 1.016, *p* ≥ 0.318). Furthermore, we did not find a significant time by treatment interaction regarding the proportion of initial saccades (*F*_2, 47_ = 0.957, *p* = 0.391), the latency of initial saccades in the long condition (*F*_2, 36_ = 0.027, *p* = 0.973) or significant higher order interactions (*F* ≤ 1.426, *p* ≥ 0.238), respectively. In addition, subsequent separate mixed models for each facial expression revealed no significant time by treatment or higher order interactions. Thus, the MAAP, NSSP and HC groups did not differ in proportions or latencies of initial saccades neither in general regarding all emotions and regions of initial fixations nor in particular regarding the eyes of angry faces.

### Hypothesis 2

To examine the second hypothesis, concerning the proposed correlation between the treatment change in behavioral and eye-tracking data assessed in the emotion classification task and the treatment change in aggressive behavior, each represented by the difference in data from pre- to post-treatment measurements (see Supplementary Table 1), we performed Pearson correlation analyses.

We only considered change in the variable response latency in the correlational analyses, as analyses with the other variables did not reveal any significant time by group interactions (see above). Table 3 presents the results of the correlational analyses.

**Table 3:** Correlational analyses between treatment change in variables measured in the emotion classification task and overt aggression measured with the OAS-M.

	**Treatment change in overt aggression scores (OAS-M, Items 1-3)**					
	**MAAP**		**NSSP**		**HC**	
	**r**	***N***	**r**	***N***	**r**	***N***
**Treatment change in response latency**						
Angry	−0.516^*^	15^a^, ^b^	−0.106	13	0.070	23
Fearful	−0.552^*^	16^a^	0.062	11^b^	0.039	22^b^
Happy	−0.571^*^	16^a^	−0.035	13	−0.097	23
Neutral	−0.406	16^a^	−0.411	13	0.138	22^b^

*OAS-M, Overt Aggression Scale Modified, Items 1–3*.

* *p < 0.05*.

a *Post-treatment OAS-M score of one patient from the MAAP group was missing*.

b *Behavioral data of one patient from each treatment group was missing*.

**Figure 2:**
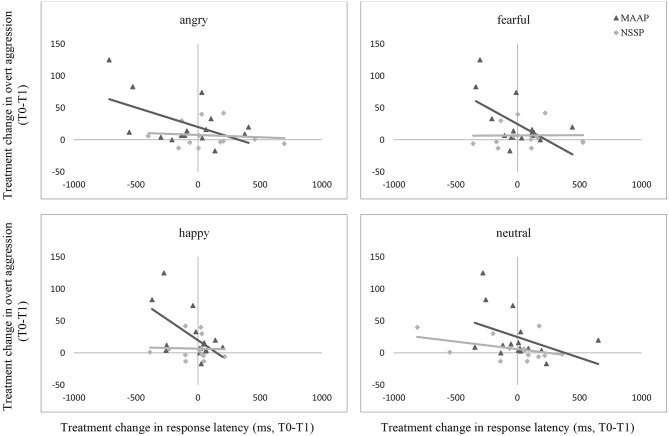
Associations between treatment change through MAAP and NSSP in overt aggression and response latency when classifying angry, fearful, happy, and neutral facial expressions. The displayed graphs represent the correlations between treatment change in overt aggression and response latency through MAAP and NSSP, respectively. T0, pre-treatment measurements; T1, post-treatment measurements; OAS-M, Overt Aggression Scale Modified, Items 1-3. Please note that data from HC is not displayed for better clarity of graphs since treatment change in overt aggression in this group was little.

The treatment change in response latency when classifying angry, fearful, and happy faces correlated negatively to the treatment change in overt aggression in the MAAP group, but not in the NSSP group or in healthy controls (see Figure 2). Thus, in the MAAP group, a reduction in aggressive behavior was related to a prolongation in the response latency when reacting to emotional facial expressions. When controlling for the pre-treatment overt aggression score, the correlations in the MAAP group stayed significant.

Hence, these findings are partially in line with our a priori hypothesis, assuming a correlation between change in emotion classification and change in aggressive behavior specifically in patients of the MAAP group, however, not only for threat-related but for all assessed emotional facial expressions.

## Discussion

This is the first study to examine changes in biobehavioral mechanisms targeted by a mechanism-based anti-aggression group psychotherapy specifically developed to reduce reactive aggression in patients with BPD. Results indicate an impact of MAAP on behavioral mechanisms of reactive aggression by showing an increase in response latency in classifying angry faces from pre- to post-treatment in the MAAP group, whereas the NSSP group showed a decrease in latency. Moreover, the change in response latencies in response to all emotional facial expressions correlated with the change in the primary outcome of the therapy study, aggressive behavior, in the MAAP group, but not in the NSSP group or in healthy controls. An increased tendency to misclassify facial expressions as angry, found at the pre-treatment time point in patients with BPD, did not change from pre- to post-treatment.

Consistent with previous studies (11, 13, 19, 21, 35), the finding that patients with BPD, but not HCs, more frequently misclassified faces as angry than other emotions in the long condition hints at a biased perception of facial expressions as angry in patients with BPD. However, contrary to our a priori hypothesis, we found no impact of MAAP on the increased tendency to misclassify facial expressions as angry in patients with BPD. Recent findings by Kleindienst et al. (36), who assessed emotion recognition ability in participants with symptom-remitted BPD, showed a persistent biased perception of facial expressions as angry compared to healthy controls, which they discussed as a trait-like feature of social cognition in BPD. This might be one reason why it seems difficult to influence the increased tendency to misclassify facial expressions as angry by a relatively brief therapeutic intervention on aggressive behavior in patients with BPD.

In line with our hypothesis, we found a change in response latencies from pre- to post-treatment, namely a prolongation of response latency when presented with angry facial expressions in the MAAP group, whereas the NSSP group, in contrast, showed a shortening of response latency. Because previous results regarding response latencies were heterogenous and both bottom-up as well as top-down processes could influence response latencies, this result can be discussed in terms of different possible mechanisms. It could reflect a change in bottom-up processes and thus hint at a reduction of the increased sensitivity to angry facial expressions in patients with BPD (37) by MAAP but not NSSP. Additionally, with regard to the extended process model of emotion regulation, in which Gross (38) describes emotion recognition (more precisely, the identification and labeling of an emotion) as a first step in emotion regulation, prolonged response latencies might be interpreted as reflecting a compensatory top-down mechanism to regulate one's emotions when presented with a highly salient social threat. Interestingly, Soler et al. (39) examined the impact of DBT on attention and impulsivity in patients with BPD compared to a clinical control group and similarly found prolonged hit reaction times from pre- to post-treatment only in the DBT group, which they interpreted as a reduction in impulsivity following DBT. Accordingly, the increase in response latency in the MAAP group might reflect an increment in cognitive control, one of the mechanisms underlying reactive aggression (5). Because in the present study, patients with BPD showed longer response latencies compared with HCs pre-treatment, the even prolonged response latencies after treatment in the MAAP group might indicate that cognitive control as a coping mechanism in dealing with aggression is enhanced by MAAP. The lack of improvement in emotion recognition accuracy, i.e., fewer misclassifications, in correspondence with this prolongation of response latencies could be explained by a ceiling effect (40), since all treatment groups already showed high accuracy in emotion recognition at baseline and, additionally, no effect of time on the proportion of misclassifications was observed regardless of treatment group.

Contrary to our a priori hypothesis, we found no effect of MAAP on the proportion or latency of initial saccades. Additionally, in contrast to a recent study using the same emotion classification task (24), which showed that patients with BPD showed more and faster initial saccades toward the eyes of briefly presented faces, we found no differences between patients with BPD and HCs in the proportion or latency of initial saccades at the pre-treatment time point. This may be due to the small sample of participants who showed initial saccades in the brief condition in the present study. Hence, our sample size might have been too limited to detect similar effects (41).

Partially consistent with our second hypothesis, we found an association between change in response latency and change in aggressive behavior from pre- to post-treatment measures, such that the longer the response latency, the greater the reduction in aggressive behavior. Importantly, this association was specific to the MAAP group and hence could indicate a relation between change in mechanisms and reduction in reactive aggression by MAAP. However, contrary to our hypothesis, the correlation was not specific for angry facial expressions but was found for angry, fearful, and happy emotional expressions. This may therefore point to the importance of cognitive control in the context of a possible generally heightened emotional sensitivity previously found in patients with BPD (42, 43). MAAP comprises several interventions that aim at taking one's time, thereby applying higher cognitive processing when evaluating socio-emotional stimuli, and thus seem to have influenced the change in response latencies: First, the app-based attention tasks focus on taking enough time to find safety cues which need effort to be identified. Second, patients are instructed to carefully question their perception of emotional stimuli and train to adequately mentalize the emotions of others (mentalization training sessions 7-11). Such processes might only be possible if the initial response provoked by threat hypersensitivity is prolonged by top-down cognitive mechanisms. Being related to a reduction in reactive aggression, as measured by the primary endpoint, the prolongation of response latencies seems to be an important reflection of potential mechanisms of change when addressing aggressive behaviors in psychotherapeutic interventions for patients with BPD. However, since prolongation of response time was not accompanied by reduction of misclassification of facial emotions, subsequent studies are needed to further deepen our understanding of the mechanisms of change and to relate them to the individual therapy modules, thus allowing for further development of MAAP or other interventions focusing on the reduction of aggressive behaviors. The lack of reduction of misclassification could also be due to a ceiling effect, and thus increasing the difficulty of the emotion classification task might be useful in subsequent studies. Finally, complementary studies on neuronal responses may provide further information on disentangling the mechanisms initiating change.

Despite the strength of investigating biobehavioral mechanisms of change in an anti-aggression group therapy specifically designed to reduce aggressive behaviors in patients with BPD, some limitations of the present study need to be acknowledged. First, since our experimental protocol was complex and included a six-week therapy for patients with BPD, as well as pre- and post-measurements, the sample of patients who participated in the post-measurements and thus could be fully included in the pre-post analysis of the behavioral mechanism was reduced compared with the sample at baseline. Hence, a potential impact of MAAP on the increased tendency of patients with BPD to misclassify facial expressions as angry might not have been detected due to the small sample size, and further replication studies with a larger sample of participants are needed to allow firm conclusions about non-significant therapeutic effects. Second, the decrease in aggressive behavior of patients with BPD from the MAAP group compared with the NSSP group reached significance only at a 6-month follow-up time point, whereas the emotion classification task was performed only at pre- and post-treatment time points. Nevertheless, in identifying mechanisms of change in psychotherapy, change in mechanisms is assumed to precede change in symptoms (10, 44), and thus it is possible to observe change in potential therapeutic mechanisms even though symptomatic change has not yet fully developed. Third, the sample of patients with BPD showed a high number of comorbid psychiatric diagnoses, so we cannot be certain that our results can be specifically attributed to BPD. Nevertheless, the number of psychiatric comorbidities observed in our study is consistent with previous studies with aggressive patients with BPD (45) and thus underlines the representativeness of our sample. Fourth, we did not account for possible fatigue effects in the emotion classification task. However, to minimize fatigue effects, we limited the block length to approximately 10 min with regular breaks in between, which is consistent with the experimental design used in previous studies (19, 24, 46).

In future studies it may also be of interest to compare groups of patients with BPD to groups of patients with other psychiatric disorders that also exhibit aggressive behavior, for instance post-traumatic stress disorder, to be able to conclude whether MAAP is tailored to the specific needs of aggressive patients with BPD or rather targets a transdiagnostic mechanism that underlies reactive aggression across various psychiatric disorders. In addition, to disentangle the different mechanisms of change, namely threat sensitivity and cognitive control, and to further differentiate between bottom-up and top-down processes, future independent studies could include additional tasks to assess the biobehavioral mechanisms more distinctly that serve as mediators of change in aggressive behavior in patients with BPD by MAAP.

In conclusion, the present findings highlight the relevance of threat hypersensitivity and cognitive control in patients with BPD as therapeutic targets and thus as potential mechanisms of change in psychotherapy aimed at reducing reactive aggression in BPD.

## Data Availability Statement

The raw data supporting the conclusions of this article will be made available by the authors, without undue reservation.

## Ethics Statement

The studies involving human participants were reviewed and approved by Ethics Committee of the Medical Faculty of the University of Heidelberg. The patients/participants provided their written informed consent to participate in this study.

## Author Contributions

SH and KB designed the study. HH, CN, KS, and MK acquired the data. HH performed the data analysis and interpreted the data together with CN, SH, and KB. HH and CN wrote the article. HH, CN, SH, KB, NK, KS, and MK critically reviewed the article. All authors approved for publication.

## Conflict of Interest

The authors declare that the research was conducted in the absence of any commercial or financial relationships that could be construed as a potential conflict of interest.

## Publisher's Note

All claims expressed in this article are solely those of the authors and do not necessarily represent those of their affiliated organizations, or those of the publisher, the editors and the reviewers. Any product that may be evaluated in this article, or claim that may be made by its manufacturer, is not guaranteed or endorsed by the publisher.
