# Supplementary material for: Impact of a Mechanism-Based Anti-Aggression Psychotherapy on Behavioral Mechanisms of Aggression in Patients With Borderline Personality Disorder
Source: Front Psychiatry. 2021 Aug 5;12:689267. doi: 10.3389/fpsyt.2021.689267 (PMC8374952; doi:10.3389/fpsyt.2021.689267)
Supplement: Supplementary file 1 [file Table_1.pdf]

Supplementary Table 1. Descriptive statistics of behavioral and eye-tracking measures (mean [m] ± one standard deviation [SD]) of patients with borderline personality disorder (BPD, randomized into MAAP or NSSP treatment) and healthy controls (HC) at pre- and post-treatment time points

	Pre-treatment (T0)			Post-treatment (T1)		
	BPD		HC	BPD		HC
	MAAP	NSSP		MAAP	NSSP	
OAS-M, Overt Aggression	38.58 (38.45)	23.06 (18.79)	0.25 (0.44)	16.38 (22.17)	15.80 (12.79)	1.04 (1.85)
Proportion of misclassifications	8.94 (16.11)	11.35 (18.24)	6.07 (10.79)	7.61 (15.06)	8.85 (14.91)	6.73 (13.31)
Angry	11.92 (13.20)	12.36 (17.56)	10.00 (12.90)	11.57 (15.19)	13.00 (14.03)	11.25 (13.63)
Fearful	9.62 (14.46)	17.50 (23.36)	9.06 (12.74)	7.86 (13.18)	8.80 (14.52)	7.79 (15.26)
Happy	1.41 (3.86)	4.31 (9.76)	1.15 (3.52)	1.71 (5.38)	2.40 (4.76)	2.21 (5.02)
Neutral	12.82 (23.84)	11.25 (17.52)	4.06 (8.65)	9.29 (20.73)	11.20 (20.17)	5.67 (15.06)
Error types	2.83 (4.77)	3.41 (5.12)	1.89 (3.07)	2.60 (5.26)	3.22 (5.81)	1.74 (2.58)
As angry	5.14 (7.60)	5.14 (5.09)	2.95 (3.69)	5.00 (9.36)	5.00 (5.63)	2.08 (2.11)
As fearful	3.69 (3.87)	4.12 (4.23)	2.74 (3.29)	2.68 (2.24)	4.27 (7.15)	2.85 (3.44)
As happy	0.77 (1.34)	1.34 (2.70)	0.35 (0.84)	0.83 (1.16)	2.00 (6.61)	0.54 (0.97)
As neutral	1.71 (2.53)	3.06 (6.93)	1.53 (2.95)	1.90 (3.13)	1.60 (1.95)	1.47 (2.63)
Response latency	1258.05 (402.91)	1271.17 (398.64)	1029.79 (270.54)	1301.57 (413.66)	1285.27 (389.41)	1053.38 (305.31)
Angry	1386.10 (411.53)	1439.78 (591.30)	1184.68 (279.07)	1494.58 (442.72)	1394.91 (347.69)	1252.85 (373.80)
Fearful	1355.61 (333.16)	1376.87 (597.33)	1172.37 (255.11)	1384.34 (361.78)	1360.56 (342.17)	1143.11 (247.09)
Happy	1056.51 (292.75)	1050.84 (389.82)	805.12 (126.36)	1080.61 (313.81)	1136.04 (315.42)	844.57 (194.26)
Neutral	1233.98 (470.77)	1217.19 (547.05)	956.99 (187.04)	1245.97 (411.27)	1249.56 (486.13)	973.20 (191.83)
Proportion of saccades	56.84 (35.33)	60.31 (35.57)	56.48 (35.18)	56.84 (34.89)	51.42 (36.75)	56.71 (35.44)
Angry	58.95 (36.58)	60.47 (33.33)	58.94 (35.36)	56.25 (34.11)	55.32 (35.60)	57.20 (34.41)
Fearful	56.60 (33.94)	62.37 (36.89)	54.78 (35.86)	53.92 (37.42)	50.69 (37.87)	58.56 (37.32)
Happy	53.94 (35.67)	58.56 (36.93)	53.99 (35.16)	58.60 (33.94)	49.09 (37.76)	54.22 (33.74)
Neutral	57.90 (35.63)	60.18 (35.67)	58.20 (34.63)	58.53 (34.57)	50.59 (36.54)	56.82 (36.51)
Saccadic latency (long condition)	354.85 (86.75)	360.16 (117.84)	364.29 (121.42)	374.65 (96.18)	381.28 (125.59)	381.36 (121.79)
Angry	349.30 (99.14)	370.65 (121.22)	364.27 (112.56)	360.61 (90.32)	370.40 (121.58)	369.25 (111.64)
Fearful	356.95 (87.30)	366.20 (125.06)	360.38 (130.16)	375.17 (85.42)	403.01 (137.33)	376.10 (110.30)
Happy	365.30 (84.56)	365.75 (135.44)	372.80 (130.49)	393.81 (111.62)	381.00 (139.66)	414.63 (154.93)
Neutral	347.83 (77.13)	338.67 (88.79)	359.97 (115.27)	368.46 (95.50)	371.24 (106.85)	365.02 (99.36)

Note. OAS-M: Overt Aggression Scale Modified; Proportion of misclassifications, error types and proportion of saccades are displayed in percent (%). Response latency and saccadic latency are displayed in milliseconds (ms). For saccadic latency, only descriptive statistics from the long condition are presented because the analysis of saccadic latencies could only be performed in this condition.
